# Comparative Study of Computerized Tomography Imaging of Facial Trauma Using 2D and 3D Reconstruction Scans

**DOI:** 10.7759/cureus.65217

**Published:** 2024-07-23

**Authors:** Aniketan KV, Shivamurthy KC

**Affiliations:** 1 Surgery, Shri BM Patil Medical College Hospital and Research Centre, Vijayapura, IND; 2 Plastic Surgery, SS Institute of Medical Sciences and Research Centre, Davanagere, IND

**Keywords:** self-fall, assault, road traffic accident, facial bone fractures, 2dct, 3dct

## Abstract

Background

The article intends to compare the efficacy of detecting fractures of facial bones in terms of the number of fractures detected, single or multiple involvement, displaced or undisplaced, segmental involvement, and comminuted or non-comminuted using 2D computerized tomography (CT) versus 3DCT for planning optimum treatment.

Methodology

One hundred patients with suspected facial bone fractures sustained either by assault, road traffic accident, or self-fall on arrival to casualty were examined clinically. Subsequently, palpation was done to detect facial bone fractures. On suspicion, they were subjected to a CT scan of the face in both coronal and axial views. The results were interpreted in the form of bones involved and detection of fractures of the same on both 2D and 3D scans. The acquired images of fractures obtained by 2D scan were reconstructed using software to obtain virtual images of the same by 3D scan to help further delineate which fractures or combination of them are better appreciated on both scans.

Result

Out of 100 patients, 52 had maxilla fractures, which were better delineated by 3D scans. The detection of zygomatic arch fractures was almost equal on both scans. Mandibular and orbital fractures were better delineated by 3D scans.

Conclusion

To conclude, we believe that in maxillofacial trauma, 3DCT provides better information than 2DCT, especially with regard to the delineation of fractures and the involvement of single or multiple bones, as it gives a real-time picture of the same. This helps in planning the management of patients, whether surgical intervention is required or can be managed conservatively.

## Introduction

The rapid pace of modern life coupled with high-speed travel as well as increased violence among intolerant society, causing facial injury with subsequent deformity either due to negligence or delay in receiving treatment due to the stigma associated with it, has become a form of social injury from which none is immune [1]. Without a strong clinical suspicion and the right diagnostic tools (CT scans with multiplanar reconstruction and panorex films), a facial fracture might not be diagnosed for a long time and might not be seen until the swelling goes down [2]. Maxillofacial injuries per se are clinically important from a surgeon’s point of view because, most of the time, there will be a combination of both soft tissue and bony involvement, which requires surgical intervention and otherwise might have serious functional and cosmetic sequelae if left untreated.

Traumatic injuries to the face pose a significant global health burden. Facial trauma, also called maxillofacial trauma, is defined as any physical injury to the face. Fractures of the face may go unnoticed if the patient has multisystem involvement. In addition, intoxicated, intubated, and sedated patients who have sustained facial fractures may go unnoticed initially, resulting in a delay in starting treatment. Early detection of these fractures is very important in deciding the plan of action, analyzing the mode of injury, and anticipating the functional and cosmetic outcomes in these patients.

The incidence and epidemiological causes of faciomaxillary trauma vary widely in different regions of the world due to social, economic, and cultural differences, in addition to awareness regarding different traffic regulations and alcohol consumption. Reports from distinct regions in Turkey also have different etiological findings [3,4]. According to the studies conducted, it has been found that assault is the leading cause of facial fractures in developed countries, followed by motor vehicle accidents, pedestrian collisions, stumbling, sports, and industrial accidents. Still, in underdeveloped or developing countries, the leading cause has shifted to road traffic accidents, followed by assault and various other reasons, one being war [5-11].

The time of maximum incidence of maxillofacial injury is usually after evening hours, which can be correlated with an increase in traffic and consumption of alcohol [12]. Maxillofacial trauma usually presents in the emergency department as an isolated injury or part of polytrauma. Restoration of facial aesthetics and function is an essential aim of recovery. Routine use of a computerized tomography (CT) scan aids in the faster diagnosis of these injuries. The primary definitive treatment of open reduction and internal fixation using titanium mini plates and screws has become the gold standard, offering optimum results [13]. Another form of CT, called cone beam CT (CBCT) scan, which provides high-resolution 3D images of the facial bones, displaying intricate details about the extent and complexity of fractures, has been in use today. This modality has a slight edge over the conventional one, especially when assessing fractures of the orbital bones, zygoma, and complex mid-face injuries. It also facilitates precise pre-surgical planning and is indispensable in evaluating potential complications, such as airway compromise, vascular injury, or cranial involvement in severe trauma cases. The capacity to visualize fractures from multiple angles with intricate detail and better quality than the conventional CT within a short period of time makes it an indispensable equipment in the diagnostic workup of facial fractures. In this study, we compared the efficacy of delineating facial fractures by both 2DCT and 3DCT scans for further planning and necessary treatment.

## Materials and methods

One hundred patients between the ages of 15 and 75 with facial bone fractures on suspicion either by assault, car accident, or self-fall on arrival to casualty were examined in detail. The diagnosis of fracture was made on palpation by the feel of crepitus. Each bone was palpated separately with the patient facing toward the clinician. The diagnosis of frontal bone fracture was done by looking at the depression of the part of the frontal region. Similarly, maxilla fracture was diagnosed by palpation along the bone to feel for any crepitus and look for signs like subconjunctival hemorrhage, diplopia, edema, etc., on the affected side. Similarly, patients with suspected nasal bone fractures were diagnosed clinically by profuse bleeding from the nose with deviation of nasal bones. Patients with suspected mandible fractures were asked to open and clench the teeth to look for any malocclusion and defined its class and diagnosed accordingly. On suspicion, they were subjected to a CT scan of the face in both coronal and axial views after stabilization in the emergency room. Patients with spine injuries who were intubated on admission and pregnant women were excluded from the study. The equipment used in this study was a Toshiba Activion 16 slice CT. The scans were taken in axial and coronal sections with the patient in the supine position. The technical specifications were as follows: exposure, 120 kVp, 130 mAS; scan time, 1.5 s; bone window, window width = 2000 HU, window level = 350 HU; thickness of the cut, 2-3 mm/s at 1:1 helical patch with a table movement speed of 2-3 mm/rotation/s. The acquired 2D images were reconstructed using 3D software. Experienced clinicians interpreted them, and they planned treatment accordingly. This observational study was conducted at a teaching institute in Karnataka. Informed consent was obtained from all patients enrolled in this study. Ethical clearance was obtained from the institutional ethical committee.

Inclusion criteria

All patients attending Plastic Surgery OPD and/or admitted with a history of either RTA, assault, or self-fall and with suspected facial bone fractures on clinical examination and those willing to undergo CT scans were included in the study, irrespective of age and sex.

Exclusion criteria

Patients with suspected facial bone fractures associated with a spine injury, patients who are intubated on admission, and pregnant women in first and second trimesters were excluded from the study.

The acquired image was put into 3D software to obtain a 3D film of the same. A single radiologist recorded, reported, and compared both 2D and 3D films separately. Details of facial bone fractures were reported. The obtained 3D images were shown to the patients and relatives, and a further management plan was discussed and decided accordingly. The images were displayed for the involvement of which bone and the number of bones involved and observed for delineation of fractures, which are easily appreciated on both 2DCT and 3DCT scans. The criteria used for the delineation of fractures were the number of bone involvement, isolated or multiple involvement, displacement or undisplacement, and, at last, comminuted or noncomminuted.

The acquired 2D images were reconstructed using 3D software. IBM SPSS Statistics, version 22.0 (IBM Corp., Armonk, NY), was used to analyze the data. Cohen’s kappa coefficient measured the agreement between the two tests. Qualitative data were represented in the form of frequency and percentage.

## Results

There were 91 men and nine women. Their ages ranged from 15 to 75 years old. All were treated using open reduction and internal fixation under general anesthesia. Road traffic accident was the most common mode of injury, followed by self-fall and assault.

Facial bone involvement

Maxilla was the most common bone, followed by the mandible, zygoma, and nasal bone. Ethmoid and hard palate fractures were the least common. 2D scanning helped detect fractures of all the walls of the maxilla with associated hemosinus, which was statistically significant, whereas 3D had little benefit in detecting all fracture walls. Even hairline fracture detection or minor deviation was better appreciated by 2D scans as they decided whether to proceed with conservative or surgical management. 3DCT helped detect only anterior wall fractures as it can detect surface fractures better, which was statistically significant. Mandibular fractures were detectable by both 2DCT and 3DCT, but precise localization of fractures was possible only by 3DCT as it could visualize their outer and inner tables. No statistical difference was noted when diagnosing fractures of the zygoma and nasal bones. However, septal fractures and hematomas were better delineated by 2D scans. Minor bone fractures like the ethmoid, sphenoid, and hard palate were better delineated by 2DCT. There was no statistical difference in detecting fractures of the lateral wall of the orbit. Le Fort fractures were better delineated by a 3DCT scan.

Based on Table 1, which shows the distribution of fractures by age group, we can conclude that the highest number of fractures occurred in the 20-29 age group, with 37 patients affected. This is followed by the 30-39 age group, with 34 patients.

**Table 1:** Distribution of fractures by age group

Age in years	Number of patients
≤20	8 (8%)
20-29	37 (37%)
30-39	34 (34%)
40-49	13 (13%)
≥50	8 (8%)
Total	100 (100%)

Based on Table 2, which presents the distribution of fractures by age and gender, we can conclude that male patients, with a total of 91 cases, significantly outnumber female patients, who account for only nine cases. This substantial difference highlights a notable gender disparity in the incidence of fractures.

**Table 2:** Distribution of fractures by age and gender

Age in years	Male	Female	Total
Less than 20	6 (6%)	2 (2%)	8 (8%)
20-29	35 (35%)	2 (2%)	37 (37%)
30-39	30 (30%)	4 (4%)	34 (34%)
40-49	12 (12%)	1 (1%)	13 (13%)
≥50	8 (8%)	0 (0%)	8 (8%)
Total	91 (91%)	9 (9%)	100 (100%)

From Table 3, it is evident that the majority of injuries were caused by road traffic accidents, accounting for 77% of cases. Self-falls were the second most common cause, representing 18% of cases, while assaults accounted for the remaining 5%.

**Table 3:** Distribution of patients by mode of injury RTA, road traffic accident

Mode of injury	Frequency
RTA	77 (77%)
Self-fall	18 (18%)
Assault	5 (5%)
Total	100 (100%)

Table 4 presents the distribution of facial bone fractures across various bones. The data highlight the frequency of fractures observed in each bone, providing insights into the prevalence and potential risk factors associated with different facial bones. From the below table, we conclude that the maxilla was most commonly bone-involved, followed by the zygoma.

**Table 4:** Distribution of facial bone fractures

Facial bone involved	Frequency
Maxilla	54 (54%)
Zygoma	24 (24%)
Nasal bone	41 (41%)
Mandible	14 (14%)
Frontal	3 (3%)
Orbit	8 (8%)
Hard palate	1 (1%)
Ethmoid	1 (1%)
Sphenoid	0 (0%)

Table 5 represents the Le Fort fractures. In our study, there were two Le Fort 1 and three Le Fort 2 fractures that were delineated more accurately by 3DCT than 2DCT, respectively.

**Table 5:** Le Fort fractures detected by 2D and 3D scans

Le Fort	2D	3D
1	0	2 (2%)
2	0	3 (3%
3	0	0 (0%)

In our study, the number of patients having isolated fractures was 68. The number of patients found to have double fractures came to 22, whereas the number of patients having triple fractures accounted for nine, and lastly, the number of patients having quadruple was found to be only one.

## Discussion

Traumatic injuries, particularly those involving the face, require high-level imaging to detect soft tissue injuries as well as fractures of bones. Imaging modalities like X-rays, although less time-consuming, may find it difficult to pick up some of the fractures because of the complex anatomy and the difficulties in obtaining high-quality images. In today’s era, a CT scan has become a widely accepted modality in the evaluation of maxillofacial trauma, as it helps in identifying facial fractures and their extent as well as assessing soft tissue injuries in a short duration of time, which also helps in planning surgical treatment [14]. With the routine use of CT scans with parallel 3D imaging, a new era has begun in the management of facial trauma.

Conventional radiography depicts anatomy in two dimensions. The depth or thickness of structures cannot be measured in these images. For a comprehensive evaluation of the morphology of a particular structure in this setting, 3D imaging is a must. This has a dramatic impact on facial injury management. Facial injuries often produce gross edema, which complicates clinical examination and obscures the underlying bony injuries by producing haziness in conventional radiographs. Whereas with 3DCT imaging, there is no need to wait for facial edema to subside, as it displays bony injuries even in the presence of it. 3DCT provides a major advantage as it can be explained to the patients and their relatives in their own language. Patients and their attendees can easily understand the nature and clinical course of the fracture by viewing 3D images when compared to conventional X-ray and 2DCT. Surface fractures are better delineated by 3DCT scan compared to 2DCT, which also helps determine whether surgical intervention is necessary for that particular patient. It also provides a real-time picture of the bone. Because the face is complicated and has thin bones on top of each other, which creates ghost artifacts, mid-face fractures are hard to diagnose on regular X-rays, which makes it easy for fractures to be misunderstood. Newer 3D reconstruction, which is fast and reliable, has become mandatory nowadays. The need for the same has been a matter of a decade for a few years.

Several studies have given controversial conclusions regarding this and mostly compare particular types of fractures. Currently, there is a paucity of studies comparing entire facial skeletons with 2D and 3D scans.

The study included 100 patients who were suspected of having facial fractures. The evaluation was done using a Toshiba Activation 16-slice CT scan. 2D scans were done in axial, sagittal, and coronal sections, along with 3D reconstruction of all the patients. The study group included patients in the age range of 16-62 years. Most belonged to 20-29 years and 30-39 years, with 37% and 34%, respectively. Male preponderance accounted for 91%, with a ratio of 9:1.

The most common mode of injury was road traffic accidents, comprising 77%, followed by falls from height and assault, comprising 18% and 5%, respectively. Shrishail et al., in their study, found RTA to be the most common cause of injury [6]. Maxilla fractures were better delineated by a 2D scan, as it could detect all the walls, which was statistically significant (P = 0.000) [15]. Associated hemosinus was visualized only on 2D scans. Slight displacement of bones or hairline fractures was better visualized by 2D scans, which helped decide whether conservative or surgical management was needed for the particular patient. In the 3D image, only the anterior wall of the maxilla fracture was better delineated, which was statistically significant. (P = 0.000; Cohen’s kappa coefficient = 1). Thus, the role of 3DCT in localizing other walls of maxilla fractures was negligible.

Mandibular fractures were the second most common fracture observed in our study. The segment-wise assessment showed a significantly higher incidence of fractures in the para symphysis and body of the mandible. The least area involved was the angle. 3D reconstruction definitely had the upper hand in accurately localizing part of the fractured bone.

The third common fracture observed was a zygoma, mainly a zygomatic arch. Here, 2DCT and 3DCT were equally efficient in diagnosing fractures of the zygoma and their displacements (P = 0.000, k = 1). The results are comparable to those of other studies [16-18].

Nasal bone involvement was seen in only 15% of cases in this study, either solo or as part of polytrauma. There was no statistically significant difference between 2DCT and 3DCT in diagnosing nasal bone fractures. Nevertheless, fractures involving the nasal septum were diagnosable only on 2D scans, which was clinically significant (P = 0.000, k = 1).

Our study included very few orbital wall fractures (8%), which were mostly in continuation with zygoma fractures involving only the lateral wall of the orbit. There was no statistically significant difference between 2DCT and 3DCT in diagnosing lateral wall of orbit fractures. Three patients with frontal bone fractures included in this study showed that both 2DCT and 3DCT were equally efficient in diagnosing frontal bone fractures. However, depressed fractures, comminute fragments, and posterior extent were better made out on 2DCT compared to 3DCT.

Thin bones of naso-orbito-ethmoid complexes were better visualized on 2DCT due to the pseudo-foramina effect and considerable bony overlap. Also, palatine fractures were better delineated by a 2DCT scan compared to a 3DCT. This study also included two Le Fort 1 fractures and three cases of Le Fort 2 fractures, which were better appreciated on 3DCT compared to 2DCT. There were no Le Fort 3 fractures. This study correlates with other studies by Shivalingam et al., who also proved the superiority of 3DCT [8].

The limitations of the above study include a small sample size, only two variables for comparison, and the outcome of surgery cannot be compared.

## Conclusions

The above study proves that CT scans (2DCT and 3DCT) are the imaging modality of choice in patients with suspected facial bone fractures. It is fast and reliable, and fractures can be confirmed within minutes. Both 2DCT and 3DCT were helpful in diagnosing facial bone fractures. However, 3DCT had an added advantage as it provides useful information, especially with regard to the extent of multiple bone fracture involvement with respect to planning management compared to 2DCT. A combination of 2DCT and 3DCT works harmoniously in deciding the optimal treatment plan in current clinical practice, which also helps the patient’s relatives understand the pathology.
